# Assessing emergency obstetric and newborn care: can performance indicators capture health system weaknesses?

**DOI:** 10.1186/s12884-017-1282-z

**Published:** 2017-03-20

**Authors:** Andrea Solnes Miltenburg, Richard Forget Kiritta, Thabea Benedicto Bishanga, Jos van Roosmalen, Jelle Stekelenburg

**Affiliations:** 1 Institute of Health and Society, Department of Community Medicine and Global Health, Faculty of Medicine, University of Oslo, Oslo, Norway; 2 Women Centered Care Project, a project of the African Woman Foundation, Magu, Mwanza Region Tanzania; 3 Department of Obstetrics and Gynaecology, Sekotoure Regional Referral Hospital, Mwanza, Mwanza Region Tanzania; 4 Magu District Council, Magu, Mwanza Region Tanzania; 5 Department of Obstetrics, Leiden University Medical Center, Leiden, The Netherlands; 6 Athena Institute, VU University, Amsterdam, The Netherlands; 7 Department of Obstetrics and Gynaecology, Leeuwarden Medical Centre, Henri Dunantweg 2, 8934 AD Leeuwarden, The Netherlands; 8 Department of Health Sciences, Community and Occupational Medicine, Global Health, University Medical Centre Groningen/University of Groningen, Antonius Deusinglaan 1, P.O. Box 196, 9700 AD Groningen, The Netherlands

**Keywords:** Emergency obstetric and newborn care, Signal functions, Resource problems

## Abstract

**Background:**

Regular monitoring and assessment of performance indicators for emergency obstetric and newborn care can help to identify priorities to improve health services for women and newborns. The aim of this study was to perform a district wide assessment of emergency obstetric and newborn care performance and identify ways for improvement.

**Methods:**

Facility assessment of 13 dispensaries, four health centers and one district hospital in a rural district in Tanzania was performed in two data collection periods in 2014. Assessment included a facility walk-through to observe facility infrastructure and interviews with facility in-charges to assess available services, staff and supplies. In addition facility statistics were collected for the year 2013. Results were discussed with district representatives.

**Results:**

Approximately 65% of expected births took place in health facilities and 22% of women with complications were treated in facilities expected to provide emergency care. None of the facilities was, however, able to perform at the expected level for emergency obstetric and newborn care since not all required signal functions could be provided. Inadequate availability of essential drugs such as uterotonics, antibiotics and anticonvulsants as well as lack of ability to perform vacuum extraction and blood transfusion limited performance.

**Conclusions:**

Performance of emergency obstetric and newborn care in Magu District was not in accordance with expected guidelines and highly influenced by lack of available resources and an insufficiently functioning health care system. Improving assessment approaches, to look beyond the signal functions, can capture weaknesses in the system and will help to understand poor performance and identify locally applicable ways for improvement.

**Electronic supplementary material:**

The online version of this article (doi:10.1186/s12884-017-1282-z) contains supplementary material, which is available to authorized users.

## Background

Emergency Obstetric and Newborn Care (EmONC) must be available and accessible to all women in order to avert maternal deaths and morbidities from the most frequent causes as haemorrhage, sepsis, eclampsia, obstructed labour and abortion complications. EmONC can also prevent fresh stillbirths and early neonatal deaths and morbidities [1].

The past decades Tanzania has given Reproductive Maternal Neonatal and Child Health (RMNCH) high priority which at policy level resulted in development of the ‘One Plan – to accelerate reduction of maternal, newborn and child deaths between 2008 and 2015’ [2, 3]. Implementation of policies, primarily the responsibility of regional and district councils, has received less attention, especially with regards to specific maternal and neonatal interventions [2, 4]. As a consequence insufficient progress has been made to reduce maternal and neonatal mortality, in particular in the Western and Lake Zone regions [2, 3].

There is no single intervention that works to achieve the desired reduction in maternal and neonatal mortality [5], as direct and underlying causes for poor maternal health and mortality are complex [6]. Along the continuum of care from pre-conception to post-birth as well as from individual-, family-, community level up to facility and policy level, packages of interventions can have great impact if implemented well [7]. Skilled care at birth and access to EmONC is of vital importance especially for those 15% of pregnant women who are expected to experience life-threatening maternal and/or neonatal complications [8].

Over the years useful process indicators have been developed to evaluate availability, accessibility and quality of EmONC [9, 10]. Regular assessment and monitoring of EmONC indicators can help programme planners in the field of RMNCH to identify priorities and needs to strengthen health services for women and their newborns [10]. The aim of this study was to perform a district-wide assessment of EmONC performance, to identify gaps and propose ways for improvement.

## Methods

### Setting

Magu District is one of eight districts of Mwanza Region of Tanzania and is bordered to the north by Lake Victoria and to the west by the city of Mwanza, which is the second largest city in Tanzania. The estimated population in 2012 was 299,759 [11]. Magu district had 46 health facilities: one government district hospital, four government health centres, 30 government dispensaries, nine private dispensaries, one faith based dispensary and one parastatal dispensary. Out of all government health facilities 31 provided antenatal, delivery and postnatal care. In terms of ownership, facilities are funded and supported by the district council of which the Council Health Management Team (CHMT) is responsible for decision-making. Main actors in the provision of maternal health care are (assistant) medical officers, clinical officers, enrolled and registered nurses/midwives, as well as medical attendants.

Since 2012 the Woman Centered Care Project (WCCP), a project of the African Woman Foundation [12–14], has been performing baseline research activities in the district and formed a partnership with the Magu District Council with the aim to improve accessibility and availability of quality maternal health care. Although focus has mainly been on antenatal care services and community involvement, the project together with members of the CHMT discussed the need for an EmONC assessment as improvements in the health situation of women in the district can only be reached when the complete system functions well along the continuum of care.

### Data collection

This study is based on two periods of data collection. A facility survey was performed at 13 dispensaries, one health centre and the district hospital in April-May 2014, including investigation of the availability of supplies and medicines to provide basic EmONC. Facilities were selected purposefully based on participation in the WCCP project locations. In December 2014 an EmONC assessment was performed including assessments at the district hospital and all four health centres in the district regarding their EmONC performance within the past three months. The research team interviewed (assistant) clinical officers or nurses in charge of the facilities. Facility visits included a walk-through to observe facility infrastructure, available services, staff and supplies. Maternal death review forms of 2013 were also reviewed. During the field visits and during meetings with different members of the CHMT and other stakeholders relevant observations were documented and discussed.

#### Data collection tools

For the facility survey in April-May a tool was developed based on the Johns Hopkins Program for International Education in Gynecology and Obstetrics (JHPIEGO) manual for monitoring birth preparedness and complication readiness [15]. The tool was digitalized using Magpi Data Collection Software [16]. Health facilities were visited to collect information on facility statistics for the year 2013, facility infrastructure, available services, equipment and supplies. The survey was conducted while facilities were awaiting their new batch of quarterly supplies. If supplies were told to be present but not able to be located or accessible during our visit, they were documented as absent. Non-functioning equipment was also recorded as not available.

For the EmONC assessment, the handbook ‘Monitoring Emergency Obstetric Care’ was used [10]. EmONC signal functions are the key medical interventions that are used to treat direct obstetric complications that cause the vast majority of maternal deaths around the globe. The seven functions of Basic EmONC are expected to be provided at health centre and dispensary level [3]. In referral district hospitals the nine functions of Comprehensive EmONC should be provided to save lives (see Table 1). For assessment of signal functions at dispensary level, focus was on availability of materials and supplies, rather than on performance in the last three months. Therefore signal function 4, manual removal of the placenta, was not included at dispensary level.

**Table 1:** Signal functions for basic and comprehensive EmONC services

Basic EmONC services	Comprehensive EmONC services
1) Administer parenteral antibiotics	Perform signal functions 1–7, plus
2) Administer uterotonic drugs	8) Perform surgery (cesarean section)
3) Administer parenteral anticonvulsants	9) Perform blood transfusion
4) Manually remove the placenta	
5) Remove retained products	
6) Perform assisted vaginal delivery	
7) Perform basic neonatal resuscitation	

#### Data collection team

The data collection team consisted of members and volunteers of the WCCP, in collaboration with their partners. For the facility survey, a volunteer nurse-midwife from Australia performed facility visits together with ASM. The EmONC assessment was performed by co-authors JvR and JS, experts of the Working Party on International Safe Motherhood and Reproductive Health in the Netherlands, both consultant obstetricians with several years of working experience in Tanzania and other sub-Sahara African countries. Facilities were notified of the visit prior to arrival. For all facility visits representatives of the District Medical Officer accompanied the team and joined in the assessment. Interviews during the facility survey were conducted with the help of a translator, for the EmONC assessments interviews were done in Kiswahili, with occasional translation.

### Data analysis

All data was entered in Microsoft Excel Version 15.0 to allow for calculation of frequency. To analyse the indicators, data collected from both the EmONC assessment and the facility survey at lower level facilities were used. Birth data was collected from registers (delivery register, admission register, operating theatre register, monthly reports) of the year 2013. If data was not accessible for the whole year, data was extrapolated from known parts of the year. To estimate the expected number of births in the district a Crude Birth Rate of 39 was used based on figures of the Demographic and Health Survey 2010, for mainland rural areas [17]. Research notes based on field visits were compared and discussed within the research team. A report of the facility survey was presented to the District Medical Officer and discussed with a Steering Committee on June 18^th^, 2014. The steering committee is overseeing project activities of the WCCP and comes together on a quarterly basis. Members included district and community representatives. After the EmONC assessment results were presented and discussed at a meeting of the CHMT during their annual planning meeting on December 6^th^, 2014.

## Results

A total of 13 dispensaries, four health centres and one district hospital were included in the facility survey. In 2013, 2504 (21%) births took place in the district hospital and 2531 (22%) births in the four health centres. In dispensaries births ranged from 50 to 296 births per year, with a mean of 100 births. Assuming similar patterns in the facilities, which were not surveyed, births in dispensaries were estimated to cover a total of 2600 (22%) births. In total these facilities covered 7635 facility births, approximately 65% of the expected births.

None of the dispensaries complied with the guidelines to provide basic EmONC and were either not able to administer one or all of the essential drugs: anti-convulsants, uterotonics or antibiotics. None of the dispensaries had complete birth kits. Five facilities were able to locate all essential neonatal resuscitation equipment. An overview of availability of essential equipment and supplies is presented in Table 2.

**Table 2:** Facility survey: availability of supplies^a^ for EmOC in 2014

Audit items		District hospital (*N* = 1)	Health centre (*N* = 1)	Dispensaries (*N* = 13)
Basic equipment	Stethoscope	Yes	Yes	10/13
	BP machine	Yes	Yes	8/13
	Fetoscope	Yes	Yes	13/13
	Thermometer	Yes	Yes	9/13
	Urine dipstick	Yes	No	1/13
	Antiseptic	Yes	Yes	8/13
	Sterile gauze	Yes	No	3/13
	Syringes	Yes	Yes	13/13
	Suture material	Yes	Yes	12/13
	IV solutions	Yes	Yes	10/13
	IV giving set	Yes	No	10/13
	Urine catheters	Yes	No	2/13
Infection prevention	Clean/Utility gloves	Yes	Yes	9/13
	Sterile gloves	Yes	Yes	12/13
	Chlorine	Yes	Yes	8/13
	Buckets	Yes	Yes	9/13
	Ability to boil water	Yes	Yes	11/13
	Sharp containers	Yes	Yes	12/13
Childbirth equipment	Scissors	Yes	Yes	11/13
	Clamps	Yes	Yes	10/13
	Cord ties	Yes	No	8/13
	Ring forceps	Yes	Yes	5/13
	Needle holder	Yes	Yes	6/13
	Container for placenta	Yes	Yes	4/13
	Macintosh	Yes	Yes	10/13
	Protective wear	Yes	Yes	7/13
	Vacuum extractor	Yes	No	0/13
	Self inflating resuscitation bag	Yes	No	10/13
	Neonatal mask	No	No	7/13
	Suction equipment	Yes	No	6/13
MVA	MVA Set	Yes	Yes	3/13
Medication	Local anaesthetics	Yes	Yes	10/13
	Oxytocin/ergometrin	Yes	Yes	11/13
	Magnesium sulphate/Diazepam	Yes	No	6/13
	Ampiciline	Yes	Yes	1/13
	Erythromycin	Yes	No	6/13
	Gentamycin	Yes	No	5/13
	Paracetamol	Yes	No	4/13
	Misoprostol	Yes	Yes	1/13
	Nevarapine	Yes	Yes	9/13
Blood tests	Hb	Yes	Yes	0/13
	RPR	Yes	Yes	0/13
	Malaria	Yes	Yes	4/13
	HIV	Yes	Yes	2/13
	Blood grouping	Yes	No	0/13

^a^Supplies or materials were available if at least one item was available and functioning at the time of the audit

Results with regards to the EmONC indicators are presented in Table 3. Three health centres did provide all signal functions for basic EmONC except administration of MgSO4 to prevent or treat eclampsia (supply problems) and performing assisted vaginal deliveries. One health centre appeared to have more problems; no manual vacuum aspirations were performed, parenteral antibiotics were not administered and newborn resuscitation was not performed. The district hospital was able to provide comprehensive EmONC but did not fully qualify due to non-availability of blood transfusions and no use of the available vacuum extractor in the past three months. Lack of blood was a contributing factor in at least three out of 22 maternal deaths and a frequent indication for referral to the regional hospital in Mwanza. Although a vacuum extractor was available and functioning, observations during the facility visit and lack of documentation on performance of vacuum extraction indicated it unlikely to be used.

**Table 3:** Indicators, results and interpretations of EmONC indicators

	Indicator	Description^a^	Acceptable level	Results	Interpretations
1	Availability of emergency obstetric care: basic and comprehensive care facilities	The availability of EmONC services is measured by the number of facilities that perform the complete set of signal functions in relation to the size of the population. When staff has carried out the seven signal functions of basic EmONC in the 3-month period before the assessment, the facility is considered to be a fully functioning basic facility. The facility is classified as functioning at the comprehensive level when it offers the seven signal functions plus surgery (e.g. caesarean) and blood transfusion.	There are at least five emergency obstetric care facilities (including at least one comprehensive facility) for every 500 000 population	1 dispensary delivers 4 out of 6 (67%), 12 dispensaries deliver 3 or less out of 6 (≤50%) signal functions for BEmOC^b^; 3 health centres deliver 5 out of 7 (71%), 1 health centre delivers 2 out of 7 (29%) of the signal functions of BEmONC; the hospital delivers 7 out of 9 (78%) of the signal functions of comprehensive EmONC.	None of the health institutions in Magu District fulfilled the full package of expected level of EmONC
2	Geographical distribution of emergency obstetric care facilities	The geographical distribution of EmOC services is calculated in the same way as the first, but it takes into consideration the geographical distribution and accessibility of facilities. It can help program planners to gather information about equity in access to services at subnational level.	See above	The health centers and dispensaries are geographically equitably distributed over the district	The health centers and dispensaries are geographically equitably distributed over the district
3	Proportion of all births in emergency obstetric care facilities	The proportion of all births in an area that take place in EmOC health facilities (basic or comprehensive). The numerator is the number of women registered as having given birth in facilities classified as EmOC facilities. The denominator is an estimate of all the live births expected in the area, regardless of where the birth takes place.	At least 90% health facility births (Target based on Sharpened One Plan II)	Population 299,759; Crude Birth Rate (CBR) 39/1000; expected deliveries 11,690. Hospital births 2504 (21%); hospital + HCs 5035 (43%); all facilities 7635 (65%).	The majority of women give birth in facilities, which are expected to provide EmONC but remain underutilized by one third of the population.
4	Meeting the need for emergency obstetric care: proportion of women with major direct obstetric complications who are treated in such facilities	Met need’ is an estimate of the proportion of all women with major direct obstetric complications who are treated in a health facility providing EmOC (basic or comprehensive). The numerator is the sum of all women treated for direct obstetric complications at emergency care facilities over a defined period, divided by the expected number of women who would have major obstetric complications, or 15% of expected births, during the same period in a specified area. The direct obstetric complications included in this indicator are: hemorrhage (antepartum and postpartum), prolonged and obstructed labour, postpartum sepsis, complications of abortion, severe pre-eclampsia and eclampsia, ectopic pregnancy and ruptured uterus.	100% of women estimated to have major direct obstetric complications are treated in emergency obstetric care facilities	Registered direct obstetric complications: 196; Caesarean sections (CS): 192 (assumption that CSs are done to treat or prevent direct obstetric complications) Expected number of direct obstetric complications: 11,690 × 0,15 = 1754 Met need = (192 + 196)/1754 × 100% = 22%	Health facilities are underutilized. 78% of women with complications are not seen in the facilities expected to provide EmONC services
5	Caesarean sections as a proportion of all births	The proportion of all deliveries by caesarean section in a geographical area is a measure of access to and use of a common obstetric intervention for averting maternal and neonatal deaths and for preventing complications such as obstetric fistula. The numerator is the number of caesarean sections performed in EmOC facilities for any indication during a specific period, and the denominator is the expected number of live births (in the whole catchment area, not just in institutions) during the same period.	The estimated proportion of births by caesarean section in the population is not less than 5% or more than 15%	All births: 11,690; Caesarean sections: 192. CS-percentage = 192/11,690 × 100% = 1.6% of all births Hospital-based CS percentage: 192/2504 × 100% = 7.7%	Health facilities are underutilized; many women who should have needed a CS did not get one, as a result of poor accessibility
6	Direct obstetric case fatality rate (CFR)	The direct obstetric case fatality rate is the proportion of women admitted to an EmOC facility with major direct obstetric complications, or who develop such complications after admission, and die before discharge.	<1%	Direct obstetric complications: 196 Maternal deaths: 22 (18 in the district hospital, 4 in the community) Direct obstetric CFR: 22/196 × 100% = 6%	Worries about quality of care in the facilities
7	Intrapartum and very early neonatal death rate	Indicator 7 is the proportion of births that result in a very early neonatal death or an intrapartum death (fresh stillbirth) in an EmOC facility. This new indicator has been proposed to shed light on the quality of intrapartum care for foetuses and newborns delivered at facilities. The numerator is the sum of intrapartum and very early neonatal deaths within the first 24 h of life occurring in the facility during a specific period. The denominator is the sum of all women who gave birth in the facility during the same period.	Standards to be determined	Fresh stillbirths (FSB) + very early neonatal deaths: 51 Hospital and health center births: 5035 Death rate: 51/5035 × 100% =1%	Low numbers of FSBs and early neonatal deaths are registered.
8	Proportion of maternal deaths due to indirect causes in emergency obstetric care facilities	The numerator of this indicator is all maternal deaths due to indirect causes in EmOC facilities during a specific period, and its denominator is all maternal deaths in the same facilities during the same period.	No standard can be set	Maternal deaths: 22; notification forms available: 16; maternal deaths classified: 15; indirect causes: 5 Proportion of indirect MD: 5/15 × 100% = 33%	Non-communicable diseases like malaria and HIV/AIDS are prevalent in the district, are important contributors to maternal mortality and are possibly inadequately prevented or treated during ANC.

^a^From the handbook ‘Monitoring Emergency Obstetric Care’ developed by AMDD, WHO, UNFPA and UNICEF, [10]

^b^Signal function 4 ‘Manual removal of placenta’ was not assessed; therefore dispensaries were assessed based on 6 signal functions

## Discussion

None of the facilities in Magu district was able to perform at the expected EmONC-level covering all signal functions. These findings are not surprising considering that the nation wide availability of basic and comprehensive EmONC facilities reduced between 2012 and 2015 [3, 18]. All facilities scored particularly poor on performance of assisted vaginal delivery. Poor clarity on who is qualified, lack of exposure and training and beliefs that higher cadres of health workers should perform such procedures contribute to almost district wide absence of performance of this signal function [1, 19]. Unavailability of facilities offering full basic and/or comprehensive EmONC is worrisome and contributes to poor outcomes, even after women have reached institutions. Previous studies in Tanzania reached similar conclusions [4, 19–22]. Negative in-facility experiences influence women’s perceptions of care and might influence future care seeking, leading to an increase in delay to seek care and preference for home birth [6].

The average facility distribution of 31 facilities per 300.000 population shows there is adequate coverage and plotting the institutions on a map shows good geographical distribution of facilities. Our estimation that 65% of births took place in health facilities is encouraging, considering the national average of skilled birth attendance of 50% [17]. However, health facilities appear underutilized as only 22% of women with expected major obstetric complications were treated in facilities and 1.6% of births were caesarean sections, similar to findings in other sub-Saharan countries [23].

Although our assessment did not include the full range of Essential Newborn Care it is important to include the ‘N’ in EmONC when conducting such assessments. Newborns have been left out of the global agenda for decades but are now increasingly being acknowledged through initiatives such as the ‘Every Newborn Action Plan’ [24]. Since fresh stillbirth and early neonatal death are usually caused by complications during childbirth [25], the ‘N’ is a vital part of EmONC assessments. Our assessment found low numbers of fresh stillbirths and early neonatal deaths assuming severe underreporting. To our surprise we did not find any documentation of neonatal deaths outside the district hospital. Monthly reporting books do not request this information, other than number of stillbirths and number of children born with Apgar score below 7 after 5 min. Difficulties in distinguishing between fresh stillbirths versus early neonatal deaths and overall poor reporting of perinatal deaths were challenges found in other studies as well [26].

Maternal deaths have been relatively stable in the district over the past years ranging from 17 to 22 registered maternal deaths [27]. Of the registered deaths in 2013, 81% occurred in the district hospital. Review of notification forms raised concerns about the quality of care [28]. Of all maternal deaths, 33% were classified due to indirect causes and could have been prevented with adequate attendance and quality of antenatal care. These findings highlight the importance of ensuring quality of service delivery across the continuum of care.

Although the EmONC indicators are widely used to assess and monitor EmONC performance, their usefulness to understand and improve the level of performance is debated [4, 26]. Additional assessment methods, beyond reliance on routinely available data, are suggested to be necessary to get a complete picture of the challenges in providing EmONC [23]. Our study shows that assessing supply levels of facilities at a given moment in time can help in gaining a sense of their ability to perform at least 6 of the 7 signal functions. This is in particular useful for facilities with low level of activity (e.g dispensaries) where assessing performance of signal functions over the past three months would not give a realistic idea of their ability to tackle emergencies due to low numbers of births.

Also for the higher-level facilities, inadequate availability of essential medicines and supplies for EmONC affects their ability to perform all signal functions and is a common problem in Tanzania [4, 29, 30]. It is important to note that this does not mean facilities and their staff are not capable or skilled to provide EmONC services. On the contrary, highly skilled health workers can be demoralized due to continuous shortages affecting work motivation and consequently quality of care [31, 32]. Assessments of EmONC should therefore increase attention to supply chain problems, including available procurement and distribution systems and accountability mechanisms.

The assessment captured other weaknesses in the system contributing to poor availability, accessibility and quality of EmONC, which are not captured in the assessment of indicators alone. Poor utilization of the partograph to monitor birth process is a contributor to poor quality of monitoring labour progress and poor outcomes. Although monitoring and management of labour using partograph is part of the signal functions for routine care [9] we argue its use is significant for quality basic EmONC and should therefore not be left aside in EmONC assessments. Insufficient training and limited health worker knowledge contribute to poor use of the partograph [33], however health facility staff reported that unavailability of stationary or funds to print partograph was a major contributing factor for their lack of use.

Similarly, availability of an ambulance for emergency transport depends on families contributing for fuel and cesarean sections could not always be performed at the district hospital due to lack of functioning generator or lack of available anesthesia equipment or blood for transfusion. Such poor organization of services and human resources indicate weaknesses in the system which show how relatively small problems, such as lack of access to stationaries, can prevent access to life saving services [34]. Preventing maternal and perinatal mortality requires a functioning health system, including adequate quality of care in its EmONC facilities. EmONC assessments should therefore be supplemented by mixed methods designs, including qualitative studies, which allow for contextual understanding of findings [26, 35].

### Limitations

Our findings should be interpreted with caution. Data collection in dispensaries was restricted to half of the available dispensaries and do, therefore, not necessarily give a complete picture of the district. However, our findings showing inadequate provision of basic EmONC services at all levels, make it very unlikely other lower level facilities are fully functioning EmONC facilities. Facility assessment in dispensaries was done prior to receiving new supplies, which should be taken into account. However re-assessment one year later after new supplies were provided did not show much improvement

Although our findings are typical for (maternal) health systems in poor rural districts in sub-Sahara Africa, our assessment was restricted to institutions in Magu District and the findings cannot be extrapolated to other districts. Neighbouring, Busega district, has no District Hospital and therefore women often rely on services of Magu District Hospital. This may have influenced the data explaining the relatively high percentage of facility births. Recall bias could have influenced data quality negatively, since we asked in-charges of health centres and district hospital about functioning of their facility in the past three months.

Data quality was limited at all levels of care. The research team observed many problems with documentation and reporting. All delivery books had missing information regarding outcome of birth and whether mother and child went home alive. Also many irregularities and inconsistencies were found between registries within facilities. At dispensary level there were no records of referral or adverse outcomes, despite staff informing us that these do occur. Poor documenting and reporting has been observed in other settings, and despite our knowledge of poor data quality, much of the decision-making is based on these poor numbers [36]. It is easy to assume there is underreporting of complications and poor outcomes, but at the same time many instances where staff do provide high quality of care also go undocumented.

## Conclusions

Although availability of health facilities expected to deliver EmONC are adequate in Magu District, none of the facilities perform all signal functions of BEmONC or CEmONC. Performance of signal functions is dependent on sufficient availability of supplies and resources as well as adequately trained and qualified staff, which can be assessed in line with EmONC indicators. In addition robust data collection systems are required for reliable assessments. Improving assessment approaches to account for local realities and contextual constraints will help to strive for improvements over time. Based on the reported results recommendations were formulated and discussed together with the Council Health Management Team. Importance of implementation of the recommendations and follow up according to the quality cycle was emphasized.

## Additional file

Additional file 1: Questionnaire Facility Audit 2015. (DOCX 550 kb)

## Abbreviations

CHMT: Council Health Management Team
EMONC: Emergency Obstetrics and Neonatal Care
JHPIEGO: Johns Hopkins Program for International Education in Gynaecology and Obstetrics
RMNCH: Reproductive Maternal Neonatal and Child Health
WCCP: Woman Centered Care Project
