# Supplementary material for: Assessing emergency obstetric and newborn care: can performance indicators capture health system weaknesses?
Source: BMC Pregnancy Childbirth. 2017 Mar 20;17:92. doi: 10.1186/s12884-017-1282-z (PMC5359823; doi:10.1186/s12884-017-1282-z)
Supplement: Additional file 1: — Questionnaire Facility Audit 2015. (DOCX 550 kb) [file 12884_2017_1282_MOESM1_ESM.docx]

**QUESTIONNAIRE Facility Audit 2015**

**QUESTIONNAIRE FACILITY IN GENERAL
Facility ID number**:

**Date of Audit:**

**Name of Evaluator:**

**Name of Ward**:

**Name of facility:**

**Name of person in charge**:

**Type of facility**

- Health Dispensary (1)

- Health Center (2)

- District Hospital (3)

- Regional Hospital (4)

- Private Clinic/Hospital (5)

- Nursing/Midwifery Teaching Institution (6)

- Other (7)

**Affiliation of facility**

- Government (1)

- Religious/Missionary (2)

- Private (3)

- Quasi-Government (4)

- Nongovernmental Organization (NGO) (5)

- Other (6)

**Do you know the date of the last supply order?**

Method of ordering supplies

- Call via mobile phone

- Send via SMS

- Send via post

- Bring in person to office

- Don't know

Date last MEDICAL SUPPLIES ordered: dd/mm/yy

Date last MEDICAL SUPPLIES delivered: dd/mm/yy

Date last MEDICINE ordered: dd/mm/yy
Date last MEDICINE delivered: dd/mm/yy

**Facility services available**

Is staff available to treat and refer clients 24 hours a day 7 days a week?

Does the facility have an active health committee? Is there community input into the running of the health facility?

Does the facility have a system for reviewing cases of maternal and prenatal deaths and/or complications on a regular basis?

Does the staff have access to facility guidelines (diagnosis, treatment and comlication management)

Does the facility have registration of stock (inventory)?

Does the facility have a hand washing policy?

Does the facility offer women to join the SMS campaign ‘Wazazi Nipendeni’?

What are women required to provide payment for?

- Registration

- ANC consultation

- Medications

- Blood tests

- Use of materials for delivery

- For services

- NONE

- Out of stock items

- Other

Are women told to prepare supplies for delivery?

Women are supposed to bring which kind of supplies?

- Mackintosh

- Basin

- Gloves

- Kitengue

- Scissor

- Gauze

- Other

Are labor companions including husbands allowed to enter ANC and labor room during examination of their relative.

Does the facility have a client waiting area with shelter

Examination room with adequate privacy

Does the facility have a water source: yes/no Type: - Well - Piped Water - Purchase water

Does the facility have electricity: yes/no Type: - TANESCO Grid - Solar - Other

How often do you experience not having access to electricity

- once a day

- once a week

- the whole day

- twice a day
Strongest mobile phone provider in area

- Vodacom

- Airtel

- Tigo

- Zantel

Is there internet at the facility

Do you have internet on your phone and does it work properly, please describe.

Does the facility have delivery bed and mattress:

Number of beds available delivery:_

Number of beds available postpartum:_

Does the facility have a toilet facility:

- Indoor with running water

- Indoor without running water

- Outdoor

**General equipments and supplies**

Does the facility have the following equipment available?

- Fetoscope

- Stethoscope

- Blood pressure cuff

- Thermometer

- Antiseptics

- Sterile gauze

- Syringes

- Suture material

- IV solutions

- Urine catheters

- Ampoules of H2O for inflating IDC balloon

- IV administration set

- Urinalysis dip sticks

- Maternal & neonatal scales

- Tape measure

- Oxygen supply

- Wall Clock

- Cooling system like fridge or gas cooling

- Other

**Record Keeping Checklist**

- ANC Card

- Clinic registers

- Partogram

- Vaccination record cards

- Apgar score cards

- ANC registration

- Birth registration

- HIV/AIDS registration

- Monthly/yearly reporting on ANC visits or births

- Other

**Infection prevention**

- Utility gloves

- Clean gloves

- HLD and/or sterile gloves

- Chlorine

- Ability to boil water

- Sharp containers

- Sterilize by boiling

- Sterilize by autoclave

- Other

**Childbirth Kit/Suture Kit**

- Scissors

- Clamps

- Cord ties

- 2 dry blankets/towels

- Ring forceps

- Needle holder

- Container for placenta

- Macintosh

- Protective wear

- Instrument to rupture membranes

- Vacuum extractor

- Forceps

- Other

**Newborn Resuscitation**

- Self inflating resuscitation bag

- Neonatal masks

- Suction equipment

**Manual Vacuum Aspiration**

- Speculum

- Manual vacuum aspiration syringe

- Cannula

- Tenaculum

**Drugs**

- Local anesthetic

- Oxytocin

- Magnesium sulfate

- Ampicillin

- Metronidazole

- Gentamicin

- Paracetamol

- Erythromycin

- Oral Polio Vaccine

- BCG vaccine

- Tetanus toxoid

- Iron/Folate tablets

- Sulfadoxine-pymethamine

- Diazapam

- Misoprostol

- Ergomitrin

- Nevarapine

- ARVs

-Has the facility performed blood transfusion in the past 3 month

-Are women referred to another facility to receive blood transfusion when needed?

-Are the following laboratory tests performed at the facility?

- Type and crossmatch

- HIV

- Hepatitis B

- Syphilis

- HB

- Malaria

- Rhesus

Are women referred to another facility to let the laboratory tests be performed when needed?

Has the facility provided cesarean section in the past 3 months?

Type(s) of Anesthesia Provided in the Last 3 Months:

- Spinal

- Local

- Ketamine

- General

If the facility provides general anesthesia, does the anesthesia machine work?

Does the facility have an operating room?

Are all key staff necessary to provide a cesarean section available 24 hours a day, 7 days a week?

Cesarean Section Kit with the Following Supplies. Check boxes for available supplies.

- Scalpel

- Scissors

- Artery forceps

- Needle holder

- Needle

- Doyen’s or pelvic retractor

- Self-retaining retractor

- Forceps, toothed

- Forceps, non-toothed

- Suction system

- Kidney basins

- Gallipots

Does the facility have security

Does the facility have a room to keep valuable things which can be locked

Does the facility have a safe

Does the facility work with computer already

Please mark this point with GPS: lattitude/longitude

Please check the power and describe how it functions:

Please check the lab tests, which lab tests have you seen:

- Type and crossmatch

- HIV
- Hepatitis B
- Syphilis
- HB
- Malaria
- Rhesus

Other comments:

**QUESTIONNAIRE HEALTH CARE WORKERS**

**Number of Staff:_**

Day Shift:_

Evening Shift:_

Night Shift:_

**Positions of staff**

- Nurse/Midwife:_

- Enrolled Nurse:_

- Auxiliary nurse/nurse aid:_

- Clinical assistant :_

- Medical attendant :_

- Community or Village Health Worker :_

- Clinical officer:_

- Doctor:_

**Qualifications**

- Certificate

- Diploma

- Degree

- Doctor

- Untrained

**Gender**: - Male:_ - Female:_

**Tasks Performed**

- Providing ANC consultations of first ANC visits

- Providing subsequent ANC consultations (after the 1st visit)

- Providing ANC education?

- Providing counseling for HIV testing during ANC visits?

- Prescribing medicines?

- Referral during ANC or during delivery?

- Perform PV (per vaginum) assessment during ANC and/or delivery

- Conducting normal deliveries?

- Conducting deliveries if complications arise?

- Conducting assisted deliveries (vacuum delivery)?

- Performing episiotomy?

- Perform episiotomy (or tears) repairs?

- Conducting postpartum checkups?

- Provide counseling on Family Planning?

**Does the facility work with other positions from the village**

- TBA

- Guard

- Community Health Worker

- No other positions from village

- Other

Please describe the way they collaborate:

**Is residency for nursing staff available?**

If you have a residency location, where is it?

- At the facility

- Close to facility

- In the village

- Other

Method for patients to contact nursing staff when facility is closed

- Phone

- Going to the staff home

- None

- Other

**QUESTIONNAIRE STATISTICS
Basic facility data**

Do you have Annual Statistics of the year 2014

Statistics by Month/Annual?

Number of ANC visits in time frame: _

No of 1st time ANC visits: _

Number of ANC visits <20w: _

Number of referrals during pregnancy: _

Reasons for Referral in ANC:

- High BP

- Pre-eclampsia

- Diabetes Gravidarum

- Prolonged rupture of membranes

- Malpresentation

- High risk pregnancy

- C/S <2y ago

- Previous stillbirth

- Age below 20 years

- Previous caesarean section

- Ten or more years since last pregnancy

- Three or more consecutive abortions

- Previous stillbirth/perinatal death (within one week)

- Intercurrent illnesses

- ≥5 pregnancies

- Height <150 cm

- Pelvic deformity

- First pregnancy at 35 or more years

- Previous caesarean section(s) or vacuum delivery

- Postpartum hemorrhage in previous deliver

- Retained placenta in the previous delivery

- Blood pressure ≥140/90 mmHg

- Hemoglobin less than 60% (8.5 gm/dl)

- Albumin in urine

- Sugar in urine

- Gestational age more than 40 weeks

- Intrauterine foetal death

- Abnormal lie after 36 weeks

- Oedema of the legs, face and hands

- Suspected twin pregnancy

- Fundal height too big or too small for gestation age

- Danger signs

- Other

Number of ANC cards available:_

Number of Women Older than 35:_

Number of Women Younger than 20:_

Number of Women with 3+ Deliveries:_

**Basic facility data: Birth**Number of births:_

Number of Spontaneous Vaginal Delivery:_

Number of live births:_

Number of stillbirths:_

Number of c-sections:_

Number of natal and directly post-partum referrals 2014:_

Reasons for Referral in Birth:

- Spontaneous rupture of membranes without labour

- Labour before 34 weeks

- More than 12 hours since labour started

- Variability of foetal heart beats (< 120 or > 160 beats per minute

- Abnormal lie or presentation of the baby

- Vaginal bleeding

- Elevated body temperature of >38° Centigrade

- Eclampsia or blood pressure 140/90 mmHg or more

- Haemoglobin less than 60% (8.5gm/dl)

- Small pelvis or big baby

- Meconium

- Retained placenta

- Severe perineal tear

- Blood loss more than 500 ml

- Other

Number of Maternal Deaths:_

Number of neonatal deaths within 7 days of birth:_

Number of twin/triplets births:_

Number of breech births:_

Number of near miss births:_

Number of women tested for HIV during ANC:_

Number of men tested for HIV during ANC:_

Number of HIV + results for women:_

Number of HIV+ results for men:_

**Comments:**
